# Effects of Source on the Nitrogen Uptake, Allocation Patterns, and Performance of Strawberry (*Fragaria × ananassa* Duch.): A ^15^N-Tracer Study

**DOI:** 10.3390/plants14020265

**Published:** 2025-01-18

**Authors:** Sirajo Salisu Jibia, Kanokwan Panjama, Chaiartid Inkham, Takashi Sato, Norikuni Ohtake, Soraya Ruamrungsri

**Affiliations:** 1 Department of Plant and Soil Sciences, Faculty of Agriculture, Chiang Mai University, Chiang Mai 50200, Thailand; ssjibia@gmail.com (S.S.J.);; 2 PhD. Horticulture Program, Department of Plant and Soil Sciences, Faculty of Agriculture Under the CMU Presidential Scholarship, Chiang Mai University, Chiang Mai 50200, Thailand; 3 Department of Agricultural Technology, Federal College of Agricultural Produce Technology, Kano 700223, Nigeria; 4 Economic Flower and Horticultural Crops Research Cluster, Chiang Mai University, Chiang Mai 50200, Thailand; 5 H. M. The King’s Initiative Centre for Flower and Fruit Propagation, Chiang Mai 50230, Thailand; 6 Multidisciplinary Research Institute, Chiang Mai University, Chiang Mai 50200, Thailand; 7 Faculty of Bioresource Sciences, Akita Prefectural University, Akita 010-0195, Japan; 8 Graduate School of Science and Technology, Niigata University, Niigata 950-2181, Japan

**Keywords:** nitrate, ammonium, nitrogen sources, uptake, distribution, ^15^N-tracer, strawberry

## Abstract

Nitrogen (N) is an essential determinant of strawberry growth and productivity. However, plants exhibit varying preferences for sources of nitrogen, which ultimately affects its use efficiency. Thus, it is imperative to determine the preferred N source for the optimization of indoor strawberry production. This study employed the ^15^N-tracer technique to investigate the effects of N sources on N uptake, distribution, and use efficiency, as well as the plants’ growth, for ‘Praratchatan 80’ strawberries in a greenhouse. Five treatments were applied: T1 (5.0 mM ^15^NO_3_^−^), T2 (2.5 mM ^15^NO_3_^−^ + 2.5 mM NH_4_^+^), T3 (5.0 mM ^15^NH_4_^+^), T4 (2.5 mM ^15^NH_4_^+^ + 2.5 mM NO_3_^−^), and T5 (N-free, control) in a completely randomized design. Significant (*p* < 0.05) differences were observed in N uptake and distribution and total N concentration among the treatments. Sole NH_4_^+^ promoted early N uptake and accelerated flowering, while NO_3_^−^ enhanced vegetative growth and later-stage nitrogen use efficiency (NUE). The application of combined NO_3_^−^ and NH_4_^+^ was most efficacious, balancing the benefits of both N forms. NO_3_^−^ treatment enhanced ^15^NUE by 46% compared to NH_4_^+^, and mixed N sources demonstrated superior and consistent ^15^NUE over time. NH_4_^+^, alone or with NO_3_^−^, expedited flowering by 20 days compared to sole NO_3_^−^ and N-free treatments. This study elucidates the importance of the sources of N in optimizing strawberry growth and flowering, providing a foundation for developing tailored N-management strategies. Future research should focus on refining mixed N application ratios and timings, exploring molecular mechanisms of N metabolism, and evaluating long-term impacts on strawberry production sustainability.

## 1. Introduction

Strawberry (*Fragaria × ananassa* Duch.) is an important horticultural fruit crop. Its fruits are revered by consumers worldwide owing to their distinctive taste, appearance, and nutritional value [1]. The total global annual farm gate value of strawberries and other temperate berry crops is approximately USD 3.7 billion, with strawberries accounting for more than 50% of this value [2]. Moreover, the global production of strawberries was 14.5 million metric tons in 2019, reflecting their widespread cultivation and consumption [3]. The major producers of strawberries include the U.S.A., Mexico, Turkey, Spain, and China, with China the leader in both area and yield [4]. Strawberries are rich in health-promoting compounds, such as antioxidants, including vitamin C, as well as polyphenols and flavonoids, which can combat oxidative stress and reduce the risk of chronic diseases such as cancer, cardiovascular diseases, and neurodegenerative disorders [5,6,7]. Strawberry fruits are rich in essential vitamins and minerals that support overall health [8]. They provide a good amount of dietary fiber, which aids in digestion and helps maintain a healthy gut [8]. Hence, the high nutritional value and adaptability of strawberries contribute to their global popularity and demand.

Nitrogen is the primary nutrient in the determination of strawberry productivity [9]. Adequate nitrogen feeding is essential for optimal strawberry plant growth indices, including leaf area, shoot and root biomass, and overall plant height. Studies have shown that nitrogen application enhances these growth parameters, leading to healthier and more robust plants [10,11]. Optimal nitrogen levels correlate with maximum fruit yield. However, insufficient or excessive nitrogen can reduce yield, indicating the need for balanced nitrogen application [10,11,12]. Nitrogen also affects various aspects of fruit quality, such as size, sugar content, acidity, and balance of phenolic compounds. For example, higher nitrogen levels can increase the total soluble sugar and anthocyanin content, enhancing the sweetness and color of the fruit [10,11,12,13].

Nitrogen availability is critical for crop production because it plays a crucial role in photosynthesis, protein synthesis, and other essential metabolic processes [14,15,16]. Nitrogen is also a critical component of chlorophyll, which captures light energy. It also affects the syntheses of photosynthetic enzymes and proteins that are essential for photosynthesis [17,18]. Nitrogen is a fundamental element in the metabolism of amino acids, which are the building blocks of proteins. Enzymes involved in nitrogen metabolism, such as nitrate reductase and glutamine synthetase, facilitate the conversion of absorbed nitrogen into amino acids, which are then used to synthesize proteins necessary for plant growth and development [19,20]. Nitrogen also enhances plant resistance to environmental stress by regulating hormone levels and other metabolic processes. Plant hormones such as cytokinin and ethylene play roles in root–shoot development and stress responses. Nitrogen availability and ethylene interactions can modulate various physiological processes, including leaf gas exchange and root architecture [21,22]. Nitrogen deficiency can lead to reduced leaf area, early senescence, and lower biomass, ultimately affecting crop yield and quality [20,23,24,25].

However, excess nitrogen application adversely affects crop production and quality, as well as the environment [26]. Understanding the mechanism of N uptake is essential for the optimization of the strawberry plant’s consumption efficiency for nitrogen [27]. Typically, plants acquire nitrogen from the soil in the form of ammonium (NH_4_^+^) and nitrate (NO_3_^−^), which are essential for overall plant growth and productivity [28,29,30,31,32,33]; some plant species prefer one source over another. This preference is typically linked to the physiological adaptation of plants to their habitat [34]. NO_3_^−^ is generally more abundant in soils and less likely to cause toxicity. It is often the preferred form for many crops because of its mobility in soil and ease of uptake [28,29,30,31,32,33]. NH_4_^+^, on the other hand, can be beneficial in small amounts but may cause toxicity at high concentrations, leading to root inhibition and shoot chlorosis [28,29,30,31,32,33]. Thus, combining nitrate and ammonium can enhance plant growth more effectively than either form alone can. This combination can improve nitrogen uptake and growth, as well as overall crop productivity [28,29,30,31,32,33,35]. An essential method for regulating the relative absorption of nitrogen is the alteration of the ratio of NH_4_^+^ to NO_3_^−^ in the plant nutrient solution [36]. For instance, in their ^15^N tracer study on hippeastrum, Inkham, Panjama, Sato, and Ruamrungsri [27] found that plants fed a combination of NH_4_^+^ and NO_3_^−^ had higher total dry weight, higher total nitrogen concentration, and broader nitrogen dispersion in the foliage.

Numerous studies have been conducted on the uptake and movement of nitrogen in plants, and some have explored the ^15^N tracer technique [27,37,38,39,40,41,42,43]. The ^15^N tracer technique is a powerful tool for investigating the absorption and transport of nitrogen in plants. It allows researchers to trace the fate of nitrogen, including its uptake by roots, translocation to leaves, and distribution within the plant [43,44]. Previous studies have suggested that the uptake and utilization of nitrogen by strawberry plants is a complex process that can be affected by the type of nitrogen source [45]. The current research provides limited insights into how different nitrogen sources, particularly ammonium (NH_4_^+^) and nitrate (NO_3_^−^), influence nitrogen uptake, distribution, and utilization in strawberry plants. This knowledge gap hinders the development of optimized fertilization strategies that can enhance strawberry yield and quality. Despite the recognized importance of nitrogen, the specific influence of different nitrogen sources on uptake and allocation patterns in strawberry plants, particularly in the ‘Praratchatan 80’ cultivar under evaporative greenhouse conditions, remains poorly understood. Thus, more research is needed to increase our understanding of the effects of nitrogen sources on nitrogen uptake and utilization in strawberry plants. Therefore, the present study aimed to evaluate the effects of these sources on the uptake and distribution of nitrogen in cultivar ‘Praratchatan 80’ strawberries under evaporative greenhouse conditions and using the ^15^N-tracer technique.

## 2. Results

### 2.1. Growth

#### 2.1.1. Growth Indices of Strawberry in Response to Varying Nitrogen Sources

The growth responses of ‘Praratchatan 80’ strawberry plants to varying nitrogen sources were evaluated in terms of leaf number, plant height, and crown formation at 8 weeks after treatment (Table 1). The results showed significant variations (*p* < 0.05) attributable to the nitrogen source.

The nitrate-only treatment (5 mM ^15^NO_3_^−^) produced plants with the highest leaf number, with about 53% more leaves compared to the control. Conversely, plants receiving mixed nitrogen sources exhibited significantly lower leaf numbers compared to the nitrate-only treatment.

Plant height (cm) was also significantly influenced by the source of the nitrogen (Table 1). While no statistically significant difference was observed among the nitrogen treatments, plants treated with 2.5 mM ^15^NH_4_^+^ + 2.5 mM NO_3_^−^ attained the greatest height of 24.60 cm. Nitrogen treatments increased plant height by 17–20% compared to the control.

#### 2.1.2. Effect of Nitrogen Source on Stolon Production

Another critical vegetative growth parameter in strawberries is stolon formation. The influence of nitrogen source on stolon production per plant in ‘Praratchatan 80’ strawberry is summarized in Table 1. Nitrogen availability significantly (*p* < 0.05) influenced the number of stolons per plant. The numbers of stolons per plant were at parity among the nitrogen treatments. The ^15^NH_4_^+^ mixed with regular NO_3_^−^ (2.5 mM ^15^NH_4_^+^ + 2.5 mM NO_3_^−^) yielded the highest number of stolons per plant, whereas the control group exhibited zero stolon production, indicating that nitrogen was necessary for stolon production.

#### 2.1.3. Fresh and Dry Weights of Strawberry Plants

The fresh and dry weights of strawberry organs at 60 days after treatment (DAT) varied significantly (*p* < 0.05) across the N treatments (Table 2). Strawberry plants supplied with 5 mM ^15^NO_3_^−^ exhibited the highest fresh and dry weights in leaves, roots, and stolons, suggesting that nitrate is an effective N source for promoting vegetative biomass accumulation. As expected, the control treatment resulted in markedly reduced fresh and dry weights across all organs, particularly in stolons, where no growth was observed. Interestingly, the mixed N treatments (2.5 mM ^15^NO_3_^−^ + 2.5 mM NH_4_^+^ and 2.5 mM ^15^NH_4_^+^ + 2.5 mM NO_3_^−^) generally resulted in intermediate biomass accumulation, compared to the single N forms. While NO_3_^−^ promoted vegetative growth, the highest fresh weight of the fruit was observed in plants supplied with 2.5 mM ^15^NO_3_^−^ + 2.5 mM NH_4_^+^, indicating a synergistic effect of mixed N sources on fruit production.

**Figure 1:**
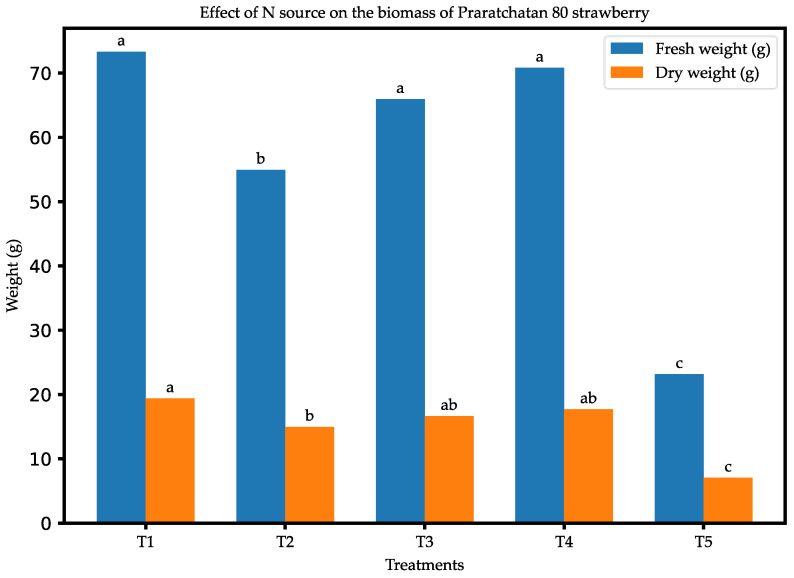
Total (a) fresh (g plant-1) and (b) dry (g plant-1) weights of strawberry plants, as affected by nitrogen sources, at 60 DAT: (1) 5 mM ^15^NO_3_^−^, (2) 2.5 mM ^15^NO_3_^−^ + 2.5 mM NH_4_^+^, (3) 5 mM ^15^NH_4_^+^, (4) 2.5 mM ^15^NH_4_^+^ + 2.5 mM NO_3_^−^, and (5) control. The different letters on the tops of the plots indicate significant differences among the treatments of varying concentration, based on the LSD test (*p* < 0.05). N = 4.

Figure 1 depicts the effects of different nitrogen sources on the total fresh and dry biomass accumulation per plant for ‘Praratchatan 80’ strawberries treated with varying nitrogen forms. The results indicated significant (*p* < 0.05) effects of the source of the nitrogen treatments on biomass production. In terms of fresh weight, the nitrate-only treatment (5 mM ^15^NO_3_^−^) produced the highest mean fresh weight (73.32 g), which was significantly higher (about 68.4%) than that of the control treatment. Similarly, significant differences were observed in the dry weights of the strawberry plants. The nitrate-only treatment produced plants with the highest dry biomass ($\bar{x}$ = 19.39 g), which was at par with those of the other nitrogen treatments. However, N-free treatment resulted in the lowest dry weight, signifying the effects of N deficiency.

### 2.2. Nitrogen Distribution and Utilization in Strawberry in Response to Nitrogen Sources

#### 2.2.1. Total Nitrogen Concentration in Strawberry Parts at 30 and 60 DAT

The total nitrogen concentration exhibited significant variations (*p* < 0.05) among strawberry plant parts at 30 and 60 days after treatment (DAT), as presented in Table 3. At 30 DAT, plants supplied with 5 mM ^15^NH_4_^+^ exhibited the highest total nitrogen concentration, significantly (*p* < 0.05) higher than all other treatments and about 94.50% more than the control. This was followed by the 5 mM ^15^NO_3_^−^ treatment. The mixed N treatments showed statistically similar total N concentrations (23.18 and 22.67 mg N g^−1^ DW, respectively).

At 60 DAT, the total nitrogen concentration decreased in all treatments except for the control group. Plants treated with 2.5 mM ^15^NH_4_ + 2.5 mM NO_3_ had the highest total nitrogen concentration, followed by those treated with 5 mM ^15^NO_3_^−^. The control group showed a significant increase (63.65%) in total nitrogen concentration compared to the value at 30 DAT. More, the plants that received the control treatment consistently displayed the lowest total nitrogen concentrations across all time-points and plant parts.

#### 2.2.2. Nitrogen Distribution (%) in Strawberry Organs

The nitrogen distribution (%) among the strawberry plant parts exhibited significant variation (*p* < 0.05) within the treatment and sampling stages (Table 4). At 30 DAT, the proportions of nitrogen allocated to leaves and roots differed significantly. The 2.5 mM ^15^NO_3_^−^ + 2.5 mM NH_4_^+^ treatment demonstrated the highest proportion of nitrogen in leaves, while the 2.5 mM ^15^NH_4_^+^ + 2.5 mM NO_3_^−^ treatment exhibited the highest root nitrogen allocation. In contrast, the 5 mM ^15^NH_4_^+^ treatment resulted in a relatively balanced nitrogen distribution between the leaves and roots.

At 60 DAT, notable shifts in nitrogen allocation patterns were observed in leaves, roots, stolons, and fruits. The 5 mM ^15^NH_4_^+^ and 2.5 mM ^15^NH_4_^+^ + 2.5 mM NO_3_^−^ treatments and the control exhibited the highest levels of nitrogen distribution to leaves (33.37%, 32.92%, and 32.64%, respectively). Concurrently, the control treatment allocated a significantly higher proportion of nitrogen to fruits than the other treatments did. The 2.5 mM ^15^NO_3_^−^ + 2.5 mM NH_4_^+^ treatment maximized root nitrogen allocation, indicating a distinct partitioning strategy, compared to the nitrate- or ammonium-only treatments. Stolon nitrogen allocation was the highest in the 2.5 mM ^15^NH_4_^+^ + 2.5 mM NO_3_^−^ treatment, while the nitrogen-free control exhibited no stolon production.

#### 2.2.3. Labeled N Concentration in Leaves, Roots, Fruits, and Stolons

The results reveal a significant difference (*p* < 0.05) in labeled nitrogen (^15^N) concentrations among the treatments across various strawberry plant parts (Table 5). At 30 DAT, the highest ^15^N concentration was recorded in the leaves of plants treated with 5 mM ^15^NO_3_^−^. Lower concentrations were observed in plants treated with 2.5 mM ^15^NO_3_^−^ + 2.5 mM NH_4_^+^ + and 2.5 mM ^15^NH_4_^+^ + 2.5 mM NO_3_^−^. When adjusted for nitrogen dose, plants treated with 2.5 mM ^15^NO_3_^−^ + 2.5 mM NH_4_^+^ exhibited the highest ^15^N concentration, followed by those treated with 5 mM ^15^NO_3_^−^. The sole application of labeled nitrate resulted in 29.44% greater ^15^N uptake compared to sole labeled ammonium, while mixing labeled nitrate with regular ammonium increased the ^15^NO_3_^−^ concentration by 62.70% compared to the reverse mixture.

At 60 DAT, the ^15^N concentration in leaves remained the highest in plants treated with 5 mM ^15^NO_3_^−^, whereas the highest concentration in fruits was observed in plants treated with 5 mM ^15^NH_4_^+^. The total ^15^N concentration was the highest in plants treated with 5 mM ^15^NO_3_^−^, followed by those treated with 5 mM ^15^NH_4_^+^. When corrected for nitrogen dose, the total ^15^N concentration was the highest in plants treated with 2.5 mM ^15^NO_3_^−^ + 2.5 mM NH_4_^+^ and 2.5 mM ^15^NH_4_^+^ + 2.5 mM NO_3_^−^, highlighting the long-term benefits of combining nitrate and ammonium over the sole application of either nitrogen form.

#### 2.2.4. ^15^N-Use Efficiency

The labeled nitrogen-use efficiency exhibited significant variation (*p* < 0.05) across treatments at 30 and 60 days after treatment (Table 6). At 30 DAT, the highest ^15^NUE was observed in 2.5 mM ^15^NO_3_ + 2.5 mM NH_4_^+^, followed by 5 mM ^15^NO_3_^−^. Conversely, T3 demonstrated the lowest efficiency, at 39.37%, indicating the reduced nitrogen-utilization efficiency observed under ammonium conditions compared with nitrate or mixed nitrogen sources.

At 60 DAT, a general decline in ^15^NUE was observed across all treatments because the labeled N application was terminated after 30 days of treatment. Plants treated with 2.5 mM ^15^NO_3_ + 2.5 mM NH_4_^+^ maintained the highest efficiency (51.04%), reflecting a superior nitrogen assimilation capacity over time, whereas sole ammonium remained the least effective. The average NUE across both sampling stages followed a similar trend, with the 2.5 mM ^15^NO_3_ + 2.5 mM NH_4_^+^ treatment exhibiting the highest overall efficiency and 5 mM ^15^NH_4_^+^ proving the lowest.

#### 2.2.5. ^15^N Concentration Decrement Percentage

Furthermore, the ^15^N decrements (%) were significantly (*p* < 0.05) different among the treatments (Table 6). As the ^15^N treatments ceased at 30 days, the differences between the ^15^N concentrations at the two time-points indicated a marked reduction. The decrease was most pronounced in plants treated with 5 mM ^15^NO_3_^−^ and lowest in 2.5 mM ^15^NH_4_ + 2.5 mM NO_3_^−^, highlighting the stabilizing effects of mixed nitrogen sources on nitrogen-utilization efficiency.

### 2.3. Flowering

#### 2.3.1. Time Taken to Flowering

The effects of nitrogen sources on the progression of blooming stages in ‘Praratchatan 80’ strawberry plants are presented in Table 7. The results indicated significant differences (*p* < 0.05) among treatments for all flowering phases.

Plants treated with ammonium-based treatments or the mixed sources of ammonium and nitrate (5 mM ^15^NH_4_^+^, 2.5 mM ^15^NH_4_^+^ + 2.5 mM NO_3_^−^, and 2.5 mM ^15^NO_3_^−^ + 2.5 mM NH_4_^+^) initiated blooming significantly earlier (about 22 days) than plants treated with nitrate alone or the control. Similar trends were observed for the time-points of 50%, 75%, and 100% bloom. Treatments with ammonium or mixed nitrogen sources consistently resulted in faster blooming progressions than those observed in nitrate-only and N-free conditions. For instance, 50% blooming was achieved in 31.13–33.25 days for ammonium-dominant treatments, whereas nitrate-only and N-free treatments required more than 52 days.

#### 2.3.2. Flowering Characteristics of Strawberry Plants

**Figure 2:**
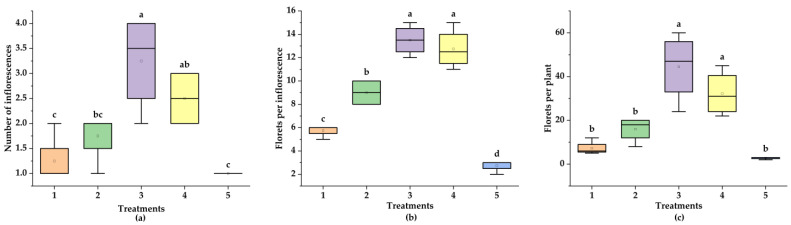
Boxplots depicting the responses of strawberry flowering attributes to varying nitrogen sources: (1) 5 mM ^15^NO_3_^−^, (2) 2.5 mM ^15^NO_3_^−^ + 2.5 mM NH4^+^, (3) 5 mM ^15^NH_4_^+^, (4) 2.5 mM ^15^NH_4_^+^ + 2.5 mM NO_3_^−^, and (5) control. ANOVA (*p* < 0.05) reveals significant differences among treatments for the following parameters: (**a**) Number of Inflorescences (F = 9.79, *p* = 0.00042); (**b**) Number of Florets per Inflorescence (F = 65.16, *p* = 2.67 × 10^−9^); (**c**) Number of Florets per Plant (F = 15.69, *p* = 3.08 × 10^−5^) N.b.: ^abc^, (e.g.) the boxplots with different superscripted letters within the same frame represent significantly different means as determined by the LSD test (*p* < 0.05).

The effects of different nitrogen sources on the flowering characteristics of ‘Praratchatan 80’ strawberry plants are shown in Figure 2. The results showed significant differences (*p* < 0.05) among treatments in terms of the number of inflorescences, number of florets per inflorescence, and total number of florets per plant. The data on the number of inflorescences and florets per inflorescence were subjected to cube root transformation.

Treatments incorporating ammonium (5 mM ^15^NH_4_^+^ and 2.5 mM ^15^NH_4_^+^ + 2.5 mM NO_3_^−^) yielded significantly (*p* < 0.05) more inflorescences than the sole nitrate or the nitrate in combination with ammonium treatments and the control, with the highest value being observed in the 5 mM ^15^NH_4_^+^ treatment (1.30). Similarly, the highest number of florets per inflorescence was observed in the 5 mM ^15^NH_4_^+^ treatment (7.63), which was significantly (*p* < 0.05) higher than those of all the other treatments. The lowest number of florets per inflorescence was observed in the control (4.10). Furthermore, the application of 5 mM ^15^NH_4_^+^ resulted in an average of 9.78 florets per plant, which was significantly higher (*p* < 0.05) than those of the other treatments.

## 3. Discussion

This study investigated the effects of varying nitrogen sources, specifically, NO_3_^−^ and NH_4_^+^ as well as their combinations, on the uptake, distribution, and utilization efficiency of nitrogen, as well as the growth and flowering of ‘Praratchatan 80’ strawberry plants under greenhouse conditions, using the ^15^N tracer technique. The results revealed that the form of the nitrogen significantly influenced all measured parameters, underscoring the critical role of nitrogen dynamics in strawberry production. This section explains the salient findings of this study in the context of the existing literature, based on the subheadings below.

### 3.1. Nitrogen Uptake and Distribution

The findings from this experiment indicated that the sole application of ammonium increased the early nitrogen concentrations in both the leaves and roots of strawberry plants. Ammonium uptake often leads to acidification of the rhizosphere, which can enhance nutrient availability and uptake in the short term. This rapid uptake is why ammonium application results in higher early nitrogen concentrations in both the leaves and roots [46]. Ammonium uptake is, in terms of energy, more efficient than nitrate uptake because it does not require reduction before assimilation into amino acids. This efficiency can lead to faster initial-growth responses [47]. This indicates the rapid action of ammonium and its suitability for topdressing applications. Nitrate, on the other hand, must be reduced to ammonium before it can be assimilated into organic compounds. Nitrate is reduced to nitrite (NO_2_^−^) by nitrate reductase (NR) and then to ammonium by nitrite reductase (NiR) [48], a process that occurs primarily in leaves and roots. Unlike ammonium, nitrate can be stored in the vacuoles. This storage capability allows plants to maintain a reserve of nitrate that can be utilized when the external supply is limited or during periods of high demand [49,50]. This delay can explain why the nitrate treatments in our experiment resulted in higher total nitrogen concentrations. It has also been reported that molecular processes such as gene expression, pH modulation, transport mechanisms, and root hydraulic conductivity can significantly enhance or deter nitrate uptake in plants [31].

Regarding the labeled N concentration, combining NO_3_^−^ and NH_4_^+^ maximized the total ^15^N (g/DW), as shown by the dose-corrected values. As nitrate is more mobile within the plant, this allows for better distribution to various plant parts over time, which could contribute to higher total nitrogen concentrations at later stages of plant growth. Moreover, combining ammonium and nitrate can maximize the efficiency of nitrogen use and enhance plant growth by balancing the benefits of both forms. The presence of both forms allows plants to optimize nitrogen uptake according to their immediate needs and environmental conditions [31,46].

Our finding that ammonium promotes early nitrogen uptake and assimilation in strawberries is corroborated by [37,46]; contrastingly, the NO_3_^−^ was more persistent and evenly distributed in plant parts, which also has been corroborated [51]. Balanced co-application of NO_3_^−^ and NH_4_^+^ has proved to be the best for many crops [31,46]. Overall, the nitrogen source and concentration exerted significant effects on nitrogen accumulation patterns in strawberry plants. Ammonium-dominated treatments promoted higher nitrogen uptake during the early growth stages but exhibited reduced partitioning efficiency at the later stages. These findings underscore the dynamic interaction between nitrogen form and plant organ-specific allocation over time. Notably, NH_4_^+^ appears to be assimilated more rapidly during the early stages, likely due to its shorter metabolic conversion pathway, which facilitates easier uptake by young plants. In contrast, NO_3_^−^ is utilized more efficiently at later stages, consistent with the well-established preference of plants for nitrate over ammonium. Further investigation is essential to determine whether targeted applications of ammonium or nitrate at specific growth stages could enhance overall growth and development in strawberry plants.

### 3.2. Nitrogen Use Efficiency

Our findings indicate that labeled nitrate-nitrogen (^15^NO_3_^−^) enhances ^15^N use efficiency (^15^NUE) by approximately 46% compared to labeled ammonium (^15^NH_4_^+^). Additionally, a drastic reduction in ^15^N concentration was observed at 60 days after treatment in the nitrate treatments, signifying a more effective and long-running nitrogen utilization in strawberry plants under nitrate-based fertilization. Increased ^15^NUE in nitrate treatments can significantly improve nitrogen use efficiency in strawberry plants, leading to reduced nitrogen loss and increased crop productivity. This is likely due to the enhanced activity of nitrate transporters and related metabolic pathways and the preference of strawberries for nitrate-N at the vegetative stage [37]. The current findings agree with [27,38], emphasizing the influence of the form of the nitrogen and concentrating on plant nitrogen uptake and retention, with mixed nitrogen treatments demonstrating a superior and more consistent ^15^NUE over time compared to the single nitrogen form.

### 3.3. Growth and Flowering Response of Strawberry to Nitrogen Sources

#### 3.3.1. Vegetative Growth Parameters

Sole nitrate application increased the number of leaves of strawberry plants by approximately 30%, compared with the sole application of ammonium. All nitrogen treatments equally increased plant height compared with that of the control. Nitrogen sources play a critical role in biomass accumulation, particularly during later stages of growth. Nitrate, as the sole nitrogen source, promoted both fresh and dry biomass production. Nitrate acts as a signaling molecule that regulates various physiological processes that are essential for plant growth and development. This includes the activation of nitrate transporters and genes involved in nitrate uptake, transport, and remobilization, which enhances nitrogen use efficiency and ultimately improves crop yields [52,53]. Nitrate generally promotes better growth and higher nitrogen use efficiency than ammonium [54]. Nitrate treatment also induces the expression of nitrate transporter genes (FaNRTs) in strawberry plants and their associated transcription factors, which are involved in nitrogen uptake and metabolism. This regulation enhances nitrogen use efficiency and promotes growth [55]. However, this effectiveness may be limited by the decreased net assimilation rate and growth inhibition by excessive nitrate quantities, compared with mixed nitrogen sources [56,57].

Mixed nitrate–ammonium treatments also supported robust strawberry growth. This synergistic effect enhances nitrogen metabolism, photosynthesis, and auxin synthesis, promotes leaf growth, and creates a large sink for carbon and nitrogen use [29,31]. These results are supported by previous findings [31,58]. The absence of nitrogen drastically reduced biomass accumulation, highlighting nitrogen’s essential role in optimizing strawberry growth and development.

#### 3.3.2. Flowering Performance

This study demonstrated significant variations in strawberry plant responses contingent upon the type of nitrogen treatment administered. The application of nitrate alone resulted in delayed flowering in ‘Praratchatan 80’ strawberry plants. Conversely, treatments incorporating ammonium, either independently or in combination with nitrate, expedited flowering by approximately 20 days relative to the nitrate-only and control treatments. Ammonium treatment had a pronounced effect on floral production. Plants subjected to ammonium treatment in isolation exhibited the highest number of florets per plant, compared to the other treatments. The observed acceleration of flowering associated with ammonium treatment was consistent with the findings of Cárdenas-Navarro et al. [59]. However, there is contrasting evidence in the literature [42]. Shi et al. [60] reported that ammonium reduced flowering in strawberry plants. These discrepancies might be explained by the faster assimilation of NH_4_^+^ than NO_3_^−^ or by cultivar-specific adaptations to different nitrogen forms. The varying responses highlight the complex interplay between nitrogen form, plant species, and flowering mechanism. However, ammonium influences the levels of plant hormones such as abscisic acid (ABA), cytokinins (CTKs), gibberellins (GAs), and indole-3-acetic acid (IAA). For example, higher ABA and CTK levels, along with lower GA and IAA levels, are associated with flower bud differentiation in strawberries [61], and might promote flowering at the early stages of strawberry growth.

## 4. Materials and Methods

### 4.1. Experimental Design, Treatments, Plant Growth Conditions, and Sampling

The experiment was conducted between November 2023 and January 2024 in an evaporative greenhouse at the King’s Initiative Center for Flower and Fruit Propagation, Ban Rai, Chiang Mai, Thailand (18° 42′ 50.148″ N, 98° 55′ 15.06″ E). The trial was laid out in a completely randomized design with five treatments and four (4) replications. Each replicate consisted of three plants. The five treatments were as follows: T1, 2.5 mM ^15^NO_3_ + 2.5 mM NH_4_^+^; T2, 2.5 mM NO_3_ + 2.5 mM ^15^NH_4_^+^; T3, 5.0 mM ^15^NO_3_; T4, 5.0 mM ^15^NH_4_^+^; and T5, N-free (control).

Young and uniform ‘Praratchatan 80’ strawberry daughter plants, previously propagated in sterile and autoclaved sand, were obtained from Wongwan Farms, in the Samoeng District, Chiang Mai. The plants were vernalized for 14 d in a growth chamber at 5 °C. The daughter plants were subsequently transferred to 7-inch plastic pots containing a rooting medium composed of vermiculite and perlite in a 2:1 ratio. This substrate blend was specifically selected to minimize the introduction of extraneous nitrogen (N) into the experiment. Both vermiculite and perlite are devoid of nitrogen, as indicated in Table 8. However, strawberry plants may still derive nitrogen contributions from endophytic associations with certain symbiotic bacteria [62]. Consequently, even in the control treatment, the nitrogen content was not an absolute zero. The plants were subsequently placed in an evaporative greenhouse under the following climatic conditions: temperature = 25 ± 2 °C, relative humidity = 75–80%, and photosynthetic photon flux density (PPFD) = 241 µmol m^−2^s^−1^.

The plants were established for two weeks before the beginning of the treatments. Each plant was then supplied with 1250 mL (15 L per 12 plants) of treatment solution (pH = 6.00) for the first 30 days. The treatment solution comprised the indicated ^15^N amount per treatment and the N-free solution based on the nutrient recipe in Table 9. The control treatment received only the N-free solution. This was followed by the application of 100 mL of CMU-S2 solution (Table 9) (pH = 6.00, EC = 1.2 dS/m) per plant per day for the remaining 30 days of the experiment.

Three sampling regimens were employed. On day zero, four plants were randomly selected as pre-treatment samples. The first sample was collected at 30 DAT. The second sampling was performed at 60 DAT (leaves, roots, stolons, and fruits per treatment). Following harvest, the strawberry plants were thoroughly cleaned with tap water to remove extraneous elements. The plants were then rinsed with deionized (DI) water to ensure comprehensive cleaning. Then, they were placed in a shaded area to allow the remaining water to evaporate naturally. Once the plants attained sufficient dryness, they were split into leaves, stolons, and roots, and their weights were determined before being transferred to an oven for additional drying, at 70 °C for 72 h. All samples were powdered and processed for further analysis.

### 4.2. ^15^N Abundance Analysis

An element analyzer (Flas 2000 HT type) and an isotope mass spectrometer (DELTA V Advantage, Thermo Fisher Scientific, Waltham, MA, USA) were used to determine ^15^N abundance and total nitrogen (%) in the strawberry plant materials. The amount of labeled N in the plants was compared to the amount of labeled N provided to determine the ^15^N use efficiency, which was expressed as a percentage [67]:

N15 use efficiency %=N15 concentration in plant N15 applied×100

The percentage distribution of total N in each organ was determined using the following equation:

$$\mathrm{Distribution}\;\left(\%\right)\;\mathrm{of}\;\mathrm{total}\;N\;\mathrm{concentration}\;=\frac{\mathrm{Total}\;N\;\mathrm{concentration}\;\mathrm{in}\;\mathrm{each}\;\mathrm{organ}\;}{\mathrm{Total}\;N\;\mathrm{concentration}\;\mathrm{in}\;\mathrm{whole}\;\mathrm{plant}}\times 100$$

The labeled nitrogen in the plants each day after treatment (DAT) was equivalent to the ^15^N concentration (in mg per plant). The labeled nitrogen supplied at each DAT time-point represents the total amount of fertilizer ^15^N applied. To make possible a direct comparison of nitrogen concentrations across treatments with differing initial application rates, the measured concentrations for the 2.5 mM ^15^N treatments were multiplied by 2 to effectively normalize the data to a 5 mM application basis.

The ^15^N increment/decrement (Δ) percentage was determined by calculating the percentage change in ^15^N concentration (in micrograms per gram dry weight) accumulated or exhausted between 30 and 60 DAT, using the following formula:

N15Δ % N15 60DAT−N15 30DATN15 30DAT×100

### 4.3. Statistical Analyses

Data analyses were conducted using the relevant Python program libraries. Statistically significant means among the treatments were separated using Fisher’s Least Significant Difference (LSD) test (*p* < 0.05).

## 5. Conclusions

The current study highlights the critical role of the source of the nitrogen in optimizing the nitrogen use efficiency, growth, and flowering of ‘Praratchatan 80’ strawberry plants under greenhouse conditions. This study also examined the nitrogen uptake, distribution, and use efficiency of strawberries by using the ^15^N tracer technique. The sole ammonium accelerated early nitrogen uptake and flower production because of the rapid and efficient assimilation of the ammonium. Conversely, nitrate treatments supported robust vegetative growth and higher nitrogen use efficiency at later stages, which are crucial for sustained development.

Moreover, these findings illustrate the dynamic interplay between nitrogen sources, plant nitrogen allocation, and crop performance. They revealed a trade-off between vegetative growth and flowering traits influenced by nitrogen form and timing. Mixed nitrogen treatments demonstrated superior nitrogen use efficiency, consistent with evidence that balanced nitrogen applications optimize plant responses to diverse growth demands and environmental conditions.

Furthermore, this study provides a foundation for tailoring nitrogen management strategies to enhance strawberry production. The findings of this study will guide the judicious application of nitrogenous fertilizers in strawberry production and form the basis for further empirical research in this domain. Future research should refine the ratios and timing of mixed nitrogen applications, explore the molecular mechanisms of nitrogen assimilation, and evaluate the long-term impacts of these treatments on soil health and crop sustainability. Such efforts will improve productivity, support sustainable agricultural practices, and ensure efficient nitrogen use in strawberry cultivation.

Lastly, this work is limited to examining the effects of nitrate-nitrogen and ammonium-nitrogen at specific concentrations—5 mM labeled NO_3_^−^ and NH_4_^+^, and 2.5 mM of each combined with 2.5 mM of the alternate nitrogen source (^14^N nitrate or ammonium)— on the ‘Praratchatan 80’ strawberry under controlled greenhouse conditions. The 10-week experiment focused on growth, flowering, and nitrogen concentrations in strawberry plant parts, without exploring molecular mechanisms or involving natural field conditions.

Future research should address these limitations by extending the experimental duration, incorporating a range of strawberry cultivars, and using field or mesocosm setups to simulate natural growth conditions. Further investigations into the molecular mechanisms that regulate nitrogen use efficiency under different nitrogen sources and combinations will provide deeper insights into the processes that influence strawberry growth and productivity.

## Figures and Tables

**Table 1:** Effects of N source on some growth parameters of ‘Praratchatan 80’ strawberry (mean ± SD).

Treatments	Number of Leaves	Plant Height (cm)	Number of Crowns	Stolons per Plant
5 mM ^15^NO_3_^−^	17.00 ± 1.07 ^a^	24.09 ± 1.46 ^a^	1.20 ± 0.53 ^a^	2.44 ± 0.38 ^ab^
2.5 mM ^15^NO_3_ + 2.5 mM NH_4_^+^	10.25 ± 0.71 ^c^	24.49 ± 0.72 ^a^	1.00 ± 0.00 ^b^	2.15 ± 0.33 ^b^
5 mM ^15^NH_4_^+^	11.88 ± 0.83 ^b^	23.66 ± 0.72 ^a^	1.00 ± 0.00 ^b^	2.18 ± 0.34 ^b^
2.5 mM ^15^NH_4_ + 2.5 mM NO_3_^−^	10.88 ± 1.00 ^c^	24.60 ± 2.25 ^a^	1.16 ± 0.52 ^ab^	2.62 ± 0.41 ^a^
Control (N-Free)	8.00 ± 0.76 ^d^	19.66 ± 2.75 ^b^	1.00 ± 0.00 ^b^	-
Sig. (*p* < 0.05)	*	*	*	*
LSD	0.90	1.80	0.34	0.54
CV (%)	7.61	7.63	14.95	15.47

N.b.: *, significant (*p* < 0.05); ^abcd^, (e.g.) the means denoted by different letters within a column are significantly different according to the LSD test (*p* < 0.05); -, no value recorded; SD, standard deviation.

**Table 2:** Fresh and dry weights of strawberry organs in response to varying N forms at 60 DAT (mean ± SD).

Treatments	Fresh Weight (g)				Dry Weight (g)		
	Leaves	Roots	Fruits	Stolons	Leaves	Roots	Stolons
5 mM ^15^NO_3_	40.32 ± 2.84 ^a^	21.99 ± 1.55 ^a^	2.08 ± 0.00 ^e^	11.00 ± 0.77 ^a^	14.30 ± 0.26 ^a^	4.78 ± 0.59 ^a^	2.34 ± 0.00 ^a^
2.5 mM ^15^NO_3_ + 2.5 mM NH_4_	30.22 ± 1.66 ^b^	16.48 ± 0.91 ^b^	5.38 ± 0.01 ^a^	8.24 ± 0.45 ^c^	13.47 ± 0.14 ^b^	2.95 ± 0.22 ^c^	1.67 ± 0.01 ^d^
5 mM ^15^NH_4_	36.27 ± 1.34 ^a^	19.78 ± 0.73 ^a^	4.90 ± 0.10 ^b^	9.90 ± 0.37 ^b^	10.86 ± 0.50 ^d^	2.95 ± 0.22 ^c^	2.26 ± 0.06 ^b^
2.5 mM ^15^NH_4_ + 2.5 mM NO_3_	38.95 ± 4.74 ^a^	21.25 ± 2.58 ^a^	4.08 ± 0.00 ^c^	10.62 ± 1.29 ^ab^	11.85 ± 0.29 ^c^	4.11 ± 0.13 ^b^	1.78 ± 0.01 ^c^
Control (N-Free)	15.05 ± 6.26 ^c^	8.11 ± 3.37 ^c^	3.82 ± 0.10 ^d^	0.00 ± 0.00 ^d^	6.16 ± 0.22 ^e^	2.59 ± 0.14 ^c^	0.00 ± 0.00 ^e^
Sig. (*p* < 0.05)	*	*	*	*	*	*	*
LSD	5.08	2.75	0.12	0.95	0.56	0.57	0.05
CV (%)	11.98	11.91	1.56	9.08	2.71	8.96	1.71

N.b.: *, significant (*p* < 0.05); ^abcde^, (e.g.) the means denoted by different letters within a column are significantly different according to the LSD test (*p* < 0.05); DAT, days after treatment; SD, standard deviation.

**Table 3:** Nitrogen concentration in strawberry parts at 30 and 60 days after treatment (mean ± SD).

Treatments	mg N g^−1^ DW at 30 DAT			mg N g^−1^ DW at 60 DAT				
	Leaves	Roots	Total	Leaves	Roots	Stolons	Fruits	Total
5 mM ^15^NO_3_^−^	40.45 ± 0.37 ^b^	39.46 ± 0.18 ^b^	40.28 ± 1.26 ^b^	21.52 ± 0.23 ^b^	21.33 ± 0.57 ^a^	17.21 ± 0.24 ^b^	12.96 ± 1.12 ^a^	20.26 ± 0.33 ^a^
2.5 mM ^15^NO_3_ + 2.5 mM NH_4_^+^	23.50 ± 0.10 ^c^	21.97 ± 0.14 ^d^	23.18 ± 0.72 ^c^	19.87 ± 0.25 ^d^	22.23 ± 0.17 ^a^	17.64 ± 0.37 ^b^	9.94 ± 0.29 ^b^	17.68 ± 0.21 ^c^
5 mM ^15^NH_4_^+^	68.16 ± 0.37 ^a^	69.24 ± 1.84 ^a^	68.36 ± 2.14 ^a^	20.93 ± 0.07 ^c^	15.79 ± 0.60 ^c^	15.33 ± 0.17 ^c^	10.67 ± 0.23 ^b^	17.17 ± 0.52 ^c^
2.5 mM ^15^NH_4_ + 2.5 mM NO_3_^−^	22.24 ± 0.47 ^c^	25.79 ± 0.69 ^c^	22.67 ± 0.71 ^c^	23.12 ± 0.15 ^a^	18.71 ± 1.20 ^b^	18.46 ± 0.59 ^a^	9.95 ± 0.29 ^b^	19.41 ± 0.23 ^b^
Control	3.71 ± 0.01 ^d^	3.90 ± 0.12 ^e^	3.77 ± 0.12 ^d^	9.99 ± 0.24 ^e^	8.18 ± 0.12 ^d^	0.00 ± 0.00 ^d^	12.44 ± 0.42 ^a^	10.37 ± 0.34 ^d^
Sig. (*p* < 0.05)	*	*	*	*	*	*	*	*
LSD	1.97	1.61	1.80	0.37	0.61	0.61	1.03	1.9
CV (%)	3.43	2.76	3.13	1.05	3.81	2.46	5.07	2.02

N.b.: *, significant (*p* < 0.05); ^abcde^, (e.g.) the means denoted by different letters within a column are significantly different according to the LSD test (*p* < 0.05); DAT, days after treatment; SD, standard deviation.

**Table 4:** Nitrogen distribution (%) in strawberry parts at different sampling stages (mean ± SD).

Treatments	30 DAT (%)		60 DAT (%)			
	Leaves	Roots	Leaves	Roots	Stolons	Fruits
5 mM ^15^NO_3_^−^	50.62 ± 0.52 ^b^	49.38 ± 0.50 ^c^	29.48 ± 0.47 ^b^	29.21 ± 0.69 ^b^	23.58 ± 0.57 ^c^	17.73 ± 0.64 ^b^
2.5 mM ^15^NO_3_ + 2.5 mM NH_4_^+^	51.70 ± 0.53 ^a^	48.30 ± 0.49 ^d^	28.51 ± 0.45 ^c^	31.91 ± 0.75 ^a^	25.32 ± 0.61 ^b^	14.26 ± 0.52 ^c^
5 mM ^15^NH_4_^+^	49.61 ± 0.51 ^c^	50.40 ± 0.51 ^b^	33.37 ± 0.53 ^a^	25.17 ± 0.59 ^d^	24.45 ± 0.59 ^b^	17.01 ± 0.62 ^b^
2.5 mM ^15^NH_4_ + 2.5 mM NO_3_^−^	47.7 ± 0.49 ^d^	52.29 ± 0.53 ^a^	32.92 ± 0.52 ^a^	26.62 ± 0.63 ^c^	26.27 ± 0.63 ^a^	14.17 ± 0.51 ^c^
Control	48.78 ± 0.50 ^c^	51.22 ± 0.52 ^b^	32.64 ± 0.52 ^a^	26.72 ± 0.63 ^c^	-	40.65 ± 1.48 ^a^
Sig. (*p* < 0.05)	*	*	*	*	*	*
LSD	0.93	0.93	0.9	1.19	0.87	1.37
CV (%)	1.03	1.02	1.58	2.35	2.4	3.63

N.b.: *, significant F-test (*p* < 0.05); ^abcd^, (e.g.) the means denoted by different letters within a column are significantly different according to the LSD test (*p* < 0.05); DAT, days after treatment; SD, standard deviation.

**Table 5:** ^15^N concentration in strawberry parts at 30 and 60 days after treatment (mean ± SD).

Treatments	mg ^15^N g^−1^ DW at 30 DAT				mg ^15^N g^−1^ DW at 60 DAT					
	Leaves	Roots	Total	Dose-Corrected	Leaves	Roots	Stolon	Fruits	Total	Dose-Corrected
5 mM ^15^NO_3_^−^	13.61 ± 0.43 ^a^	10.86 ± 0.46 ^a^	13.11 ± 0.03 ^a^	13.11 ± 0.30 ^b^	4.32 ± 0.06 ^a^	3.55 ± 0.11 ^a^	2.19 ± 0.03 ^a^	0.23 ± 0.29 ^c^	3.58 ± 0.12 ^a^	3.58 ± 0.09 ^b^
2.5 mM ^15^NO_3_ + 2.5 mM NH_4_^+^	8.17 ± 0.09 ^c^	4.56 ± 0.11 ^c^	7.41 ± 0.40 ^c^	14.82 ± 0.80 ^a^	2.30 ± 0.07 ^c^	1.91 ± 0.01 ^c^	1.43 ± 0.09 ^b^	1.46 ± 0.03 ^b^	2.00 ± 0.07 ^c^	3.98 ± 0.10 ^a^
5 mM ^15^NH_4_^+^	9.85 ± 0.31 ^b^	6.42 ± 0.55 ^b^	9.25 ± 0.18 ^b^	9.25 ± 0.18 ^c^	3.89 ± 0.11 ^b^	2.56 ± 0.12 ^b^	2.12 ± 0.04 ^a^	2.91 ± 0.09 ^a^	3.28 ± 0.11 ^b^	3.28 ± 0.08 ^c^
2.5 mM ^15^NH_4_ + 2.5 mM NO_3_^−^	5.60 ± 0.17 ^d^	3.50 ± 0.13 ^d^	2.84 ± 0.15 ^d^	5.67 ± 0.30 ^d^	2.25 ± 0.01 ^c^	1.85 ± 0.11 ^c^	1.07 ± 0.02 ^c^	1.66 ± 0.07 ^b^	1.97 ± 0.07 ^c^	3.94 ± 010 ^a^
Sig.	*	*	*	*	*	*	*	*	*	*
LSD	0.53	0.69	0.52	0.87	0.136	0.18	0.09	0.29	0.18	0.18
CV (%)	3.04	5.8	3.39	4.31	2.26	3.88	2.98	9.94	3.48	2.58

N.b., *, significant (*p* < 0.05); ^abcd^, (e.g.) the means denoted by different letters within a column are significantly different according to the LSD test (*p* < 0.05); DAT, days after treatment; SD, standard deviation.

**Table 6:** Labeled nitrogen use efficiency of strawberry at 30 and 60 days after treatment (mean ± SD).

Treatments	^15^N-Use Efficiency (%)			^15^N Decrement (%)
	30 DAT	60 DAT	Average	
5 mM ^15^NO_3_^−^	87.91 ± 1.45 ^b^	37.00 ± 1.27 ^b^	62.46 ± 0.83 ^b^	−57.89 ± 1.76 ^d^
2.5 mM ^15^NO_3_ + 2.5 mM NH_4_^+^	91.42 ± 1.26 ^a^	51.04 ± 0.47 ^a^	71.23 ± 0.74 ^a^	−44.16 ± 0.77 ^c^
5 mM ^15^NH_4_^+^	39.37 ± 1.84 ^d^	27.77 ± 0.30 ^d^	33.57 ± 1.04 ^d^	−29.39 ± 2.83 ^b^
2.5 mM ^15^NH_4_ + 2.5 mM NO_3_^−^	44.07 ± 0.88 ^c^	33.09 ± 0.52 ^c^	38.58 ± 0.63 ^c^	−24.91 ± 1.20 ^a^
Sig. (*p* < 0.05)	*	*	*	*
LSD	2.64	1.4	1.55	3.41
CV (%)	2.13	1.99	1.6	−4.64

N.b.: *, significant (*p* < 0.05); ^abcd^, (e.g.) the means denoted by different letters within a column are significantly different according to the LSD test (*p* < 0.05); DAT, days after treatment; SD, standard deviation.

**Table 7:** Time taken to initiate and progress through blooming stages, for ‘Praratchatan 80’ strawberry plants treated with varying nitrogen sources (mean ± SD).

Treatments	Days to Blooming			
	Initial	50%	75%	100%
5 mM ^15^NO_3_^−^	46.13 ± 2.59 ^a^	53.13 ± 2.57 ^a^	58.13 ± 2.54 ^a^	66.13 ± 2.34 ^a^
2.5 mM ^15^NO_3_ + 2.5 mM NH_4_^+^	24.25 ± 1.58 ^b^	31.25 ± 1.57 ^b^	36.25 ± 1.58 ^b^	44.25 ± 1.61 ^b^
5 mM ^15^NH_4_^+^	24.13 ± 1.89 ^b^	31.13 ± 1.88 ^b^	36.13 ± 1.76 ^b^	44.13 ± 1.89 ^b^
2.5 mM ^15^NH_4_ + 2.5 mM NO_3_^−^	26.75 ± 1.67 ^b^	33.25 ± 1.61 ^b^	38.38 ± 1.77 ^b^	46.13 ± 1.54 ^b^
Control	46.38 ± 4.17 ^a^	52.25 ± 4.17 ^a^	57.25 ± 4.52 ^a^	65.25 ± 4.21 ^a^
Sig. (*p* < 0.05)	*	*	*	*
LSD	2.62	2.60	2.63	2.60
CV (%)	7.70	6.44	5.60	4.82

N.b.: *, significant (*p* < 0.05); ns, not significant; ^ab^, (e.g.) the means denoted by different letters within a column are significantly different according to the LSD test (*p* < 0.05); SD, standard deviation.

**Table 8:** Approximate elemental compositions of vermiculite and perlite (weight percentages).

Element/Oxide	Vermiculite (%)	Perlite (%)
Mg	8.68	0.00
MgO	14.39	0.45
Al	23.01	0.00
Al_2_O_3_	43.48	13.50
Fe	9.97	0.00
FeO	12.82	1.25
Si	5.57	0.00
SiO_2_	11.92	72.50
H	2.00	0.00
H_2_O	17.87	4.00
O	50.77	0.00
CaO	0.00	1.00
K_2_O	0.00	4.00
Na_2_O	0.00	3.50

Adapted from [63,64,65,66].

**Table 9:** Concentrations of essential nutrients (mg/L) in CMU-S2 and the N-free nutrient recipe used in the experiment.

Nutrient Elements	CMU-S2 (mg/L)	N-Free Recipe (mg/L)
Major element		
N	147.85	-
P	39.75	55.75
K	255.31	70.39
Mg	28.51	30.29
Ca	73.23	40.65
S	38.44	60.00
Minor element		
Zn	1.37	0.60
Mn	0.61	0.25
B	1.05	0.60
Mo	0.18	0.045
Fe	3.99	4.00
Cu	0.23	0.09

N.b., CMU: Chiang Mai University.

## Data Availability

All data upon which the conclusions of this study are based are made available upon request. Requests should be directed to the corresponding authors.
